# COVID‐19 outbreak: An experience to reappraise the role of hospital at home in the anti‐cancer drug injection

**DOI:** 10.1002/cam4.3682

**Published:** 2021-03-05

**Authors:** Bénédicte Mittaine‐Marzac, Arsene Zogo, Jean‐Christophe Crusson, Valerie Cheneau, Marie‐Claire Pinel, Marie‐Laure Brandely‐Piat, Fatma Amrani, Laurent Havard, Elisabeth Balladur, Taina Louissaint, Laurence Nivet, Joel Ankri, Philippe Aegerter, Matthieu De Stampa

**Affiliations:** ^1^ Hospitalisation à Domicile Assistance Publique des Hôpitaux de Paris Paris France; ^2^ Hôpital Paul Brousse CESP ‐ Centre de recherche en Epidémiologie et Santé des Populations ‐ U1018 INSERM Université Paris‐Saclay (UPS Université de Versailles Saint‐Quentin (UVSQ Paris France; ^3^ Cochin Hospital ‐ Assistance Publique des Hôpitaux de Paris Paris France

**Keywords:** cancer, COVID‐19‐Organisation, home‐based hospital, pandemic

## Abstract

**Background:**

The COVID‐19 outbreak has posed considerable challenges to the health care system worldwide, especially for cancer treatment. We described the activity and the care organisation of the Hospitalisation At Home (HAH) structure during the pandemic for treating patients with anti‐cancer injections.

**Methods:**

We report the established organisation, the eligibility criteria, the patient characteristics, the treatment schemes and the stakeholders’ role during two 5‐week periods in 2020, before and during the French population's lockdown.

**Results:**

The increase of activity during the lockdown (+32% of treated patients, +156% of new patients and +28% of delivered preparations) concerned solid tumour, mainly breast cancer, even if haematological malignancies remained the most frequent. Thirty different drugs were delivered, including three new drugs administered in HAH versus 19 during the routine period (*p* < 0.01). For those clinical departments accustomed to using HAH, the usual organisation was kept, but with adjustments. Five clinical departments increased the number of patients treated at home and widened the panel of drugs prescribed. Three oncology departments and one radiotherapy department for the first time solicited HAH for anti‐cancer injections, mainly for immunotherapy. We adjusted the HAH organisation with additional human resources and allowed to prescribe drugs with an infusion time of <30 min only for the new prescribers.

**Conclusion:**

HAH allowed for the continuation of anti‐cancer injections without postponement during the pandemic, and for a decrease in unnecessary patient travel to hospital with its concomitant COVID‐19 transmission risk. Often left out of guidelines, the place of HAH in treating cancer patients should be reappraised, even more so during a pandemic.

## Graphical abstract

HAH could be used to decrease unnecessary travel to hospital and allow further anti‐cancer treatments and follow‐up from home particularly during the pandemic period. HAH should be included in guidelines for cancer administration and not restricted to follow‐up or oral treatment. It is necessary to rethink the classical cancer management scheme and develop alternative models of health service delivery such as home programs to manage this public health issue in both the short‐ and also long‐term.

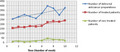

**Abbreviations**
COVID‐19: coronavirus disease 2019
HAH: Hospitalisation At Home
IQR: interquartile range
NS: non‐significance

## 1 BACKGROUND

In December 2019, an outbreak of a new coronavirus disease 2019 (COVID‐2019) spread in Wuhan, a city in the Chinese province of Hubei. It was caused by severe acute respiratory syndrome coronavirus 2 (SARS‐CoV‐2) and, though mild in most cases, represented a potentially fatal disease.^1^ COVID‐19 spread mainly through close contact from person‐to‐person in respiratory droplets. It rapidly became a major international issue.^2^ On 11 March 2020, the World Health Organisation officially declared the COVID‐19 outbreak a pandemic.^3^ As there was neither a vaccine nor a specific treatment to limit the COVID‐19 outbreak, social distancing and reduction of face‐to‐face contact were required to slow down disease transmission. Despite those measures, the worldwide health care systems were rapidly overwhelmed. On March 16th 2020, the French government declared a lockdown for the French population. Health care facilities and clinicians should prioritise urgent and emergency visits, and postpone chronic disease management to reduce pressure on health services and the risk of transmission. According to the Chinese experience, patients with cancer may have a higher risk of contracting COVID‐19 and developing complications, due either to the immunosuppressed condition linked to the disease or to the anti‐cancer treatment.^4^ In this context, guidelines were established to protect patients undergoing cancer treatment from COVID‐19 infection, such as the reduction of hospital visits, adjustment of dosing schedules of anti‐cancer treatment or the replacement of intravenous by oral medicine when possible.^5^, ^6^ However, switching the route of administration or postponing treatment is not always possible, and delays could lead to tumour progression or worsen the performance status.^7^ When ‘Home’ is mentioned in the guidelines on how to treat cancer patients during the COVID‐19 pandemic, ‘Home’ is considered suitable only for follow‐up or for the use of elastomeric pumps delivered in outpatient clinics.^4^, ^6^, ^8^

Moreover, hospital remains the main setting for parenteral cancer chemotherapy administration, despite patients’ generally positive feeling about receiving care at home.^9^

Hospitalisation At Home (HAH) is a service that can avoid hospital stays through the provision of treatment by health care professionals at home for conditions that otherwise would require care in hospital.^10^ Hospitalisation At Home provides complex care and services are available 24 h a day, 7‐days a week. Although most patients with anti‐cancer treatment used to receive their chemotherapy in a conventional hospital setting, HAH represents an alternative solution by providing care, including parenteral drug injection, at home. Despite its feasibility^11^, ^12^ and policies to promote the development of anti‐cancer treatment at home in some countries,^13^ injection of anti‐cancer drugs remains low. The COVID‐19 pandemic could be a strong argument for health care reorganisation, and for including HAH in the cancer patient's pathway. We report our experiment in the HAH of APHP, where 916 patients, adults and children, received anti‐cancer injection at home in 2019. Based on our experience of anti‐cancer treatment at home, we aimed to describe the activity and the care organisation of treating patients with anti‐cancer injections at home as an alternative to hospital during the COVID‐19 pandemic.

## 2 METHODS

### 2.1 Study design and intervention

Hospitalisation At Home (HAH) has been part of the French health system for several decades and has become relatively more important over the past 10 years. HAH’s mission is 'to ensure, for those suffering from severe, complex and progressive disease(s), the continuous and coordinated medical and paramedical care that only a hospital facility can provide, within the patient's home, for a limited period of time, depending on the evolution of the health condition'.^14^ Hospitalisation At Home provides acute care and services are available 24 h a day, 7‐days a week.^15^ This study was conducted in APHP’s HAH named HAH APHP. This structure is organised through the largest conglomerate of 37 public hospitals located in APHP and its suburbs. HAH APHP is the oldest structure of home care in France, running since 1957. It takes care of about 850 patients a day, including about 30% with cancer. Its intervention area is localised in APHP and its inner suburbs. Patients were eligible if the care home was localised in the intervention area of HAH APHP.

We analysed two periods of 5‐week activity in HAH APHP, ‘period 1’ from 17 February to 22 March, before lockdown, and ‘period 2’ from 23 March to 26 April^+^ after the beginning of the French population's lockdown. Patients were treated according to current standards of care and practice. Patients were eligible if they received at least one anti‐cancer injection during the period studied.

The administration of parenteral anti‐cancer drugs is currently performed by about 250 qualified nurses at the patient's home. Prescribers are spread across hospitals whether from APHP or not, so the link with HAH is maintained through the HAH coordination nurse, the HAH coordinator doctor and the HAH specialised pharmacist. Anti‐cancer drugs are eligible for administration in our structure based on a stability of the preparation of >8 h, an infusion time of <2 h and drug tolerance. Drugs potentially causing immediate infusion‐related reactions are excluded or require several injections in hospital before administration at home. Since 2015 every step of the medication system is computerised with the CHIMIO® information system (Computer Engineering®, France), from the medical order by a physician to the HAH nurse's administration. According to the number of hospital prescriptions for the HAH, the prescription can be recorded in the CHIMIO software in three different ways; either the physician makes the prescription on his own CHIMIO software and the order is transmitted to the HAH structure; or the physician makes the prescription directly into the HAH CHIMIO software; or a HAH coordinator doctor transcribes the order into the HAH CHIMIO software.

Once prepared, drugs are delivered daily straight to the patient's home in a sealed temperature controlled cool box by a secure carrier from the ‘Chemo unit’. Then, one of our dedicated nurses goes to the home and supervises the administration of chemotherapy. Nurses are physically present at home during injection. After injection, HAH nurses report the administration and any incidents in the CHIMIO® software. In cases of acute tolerance issues, HAH nurses call the HAH coordinator doctor, physicians or the emergency services depending on severity. As a matter of routine patients gave their informed written consent to be treated at home and to the use of their anonymised data for research.

The study was submitted to the CERAPHP5 ethics committee, but did not require approval as it was monocentric and retrospective, and patients were treated according to standard clinical practice, including no additional interventions, and the data were anonymous, with no identifiable patient information. However, the study has been recorded in the general register of APHP according to data protection legislation (n° 20200715132522).

### 2.2 Patients

The first injection of chemotherapy was always administered in the hospital to assess patient susceptibility to acute severe adverse events. According to our usual procedures, prior to each course of treatment, patients were evaluated by a physician in an outpatient clinic. During this assessment, patients underwent complete physical examinations along with appropriate blood test analysis and functional exploration––if required––to determine their eligibility for anti‐cancer treatment.

If a patient was approved for treatment, the patient had the second and subsequent cycles of chemotherapy at home except for the first administration of each course in an outpatient clinic. The HAH coordinator nurse met the patient during the first administration to assess the home environment and the patient's understanding of the HAH organisation. The patient is asked to nominate a person close to them who would be most involved in helping to support them at home.

During the course, dose modifications were defined by the physician according to blood results and clinical status.

### 2.3 Data collection

Data collection was based on the CHIMIO® software (Computer Engineering®, France). The patients’ characteristics (age, sex), hospital prescribers, department prescribers, new therapeutic protocols, drug regimen, treatment duration in HAH, number of patients and new patients and the number of anti‐cancer preparations delivered were reported during the two periods and for each week.

### 2.4 Outcomes

We described activity, care and the logistical organisation required to manage ongoing anti‐cancer treatment during the COVID‐19 pandemic.

### 2.5 Statistical methods

Data were expressed as a percentage for qualitative variables, and in median and interquartile range (IQR) [25%–75%] for quantitative variables. Comparisons of the distributions of the qualitative variables used the Pearson's chi‐squared test. Comparisons of continuous quantitative or ordinal data were performed with a Student *t*‐test. A *p* < 0.05 was considered significant. The statistical programme R Studio 1.2.5001 was used for the analysis.

## 3 RESULTS

### 3.1 Patients, drugs and therapeutic protocols

During ‘period 1’, 365 patients including 62 new patients (17%) received at least one anti‐cancer injections at home. Patients were mainly adults, with 54% men with haematological malignancies (94%). Children represented 3% of the patients. During ‘period 2’, the number of patients increased by 30%, with 473 patients treated at home, including 159 new patients (+156%). Children represented 4.4% of the patients during ‘period 2’ (Non‐significant (NS) difference). The median adults’ age was 73 years old [64;79] and the median children’ age was 10 years old [3;11] during ‘period 1’ versus, respectively 72 years [60;78] (*p* < 0.01) and 4 years [3;11] during ‘period 2’ (NS).

The number of females treated increased significantly from 46% to 55% during period 2 (*p* = 0.01). Patients were mainly treated at home for haematological malignancies with a significant increase of solid tumours from 6% to 20.9% in period 2 (*p* < 0.01).

During both periods patients were mainly treated for multiple myeloma, acute myeloid leukaemia and myelodysplastic syndrome. However, breast cancer was four times more frequent during ‘period 2’ (Table 1).

**TABLE 1:** Patients’ characteristics during the two periods

	‘Period 1’	‘Period 2’	Evolution rate (%)	*p*‐value
Number of patients (n,%)	365	473	30	
New patients	62 (17.0)	159 (33.6)	156	0.01
Adults	354 (97.0)	452 (95.6)	27,7	ns
Sex (Female)	168 (46.0)	260 (55.0)	54,8	0.01
Age (median, IQR)				
Adults	73 [64;79]	72 [60;78]	/	0.01
Children	10 [3;11]	4 [3;11]		ns
Haematological malignancy (n,%)	343 (94.0)	374 (79.1)	9	0.01
MM	126 (34.5)	150 (31.7)	19	0.05
AML and MDS	172 (47.1)	172 (36.4)	0	
ALL	11 (3)	26 (5.5)	136	
NHL and Hodgkin disease	34 (9.3)	26 (5.5)	−23.5	
Solid tumour (n,%)	22 (6.0)	99 (20.9)	350	0.01
Breast cancer	16 (4.4)	86 (18.2)	437	0.1
NSCLC and Others	6 (1.6)	13 (2.7)	117	

IQR, Interquartile range [+25%‐+75%]; ns, non‐significant; MM, multiple myeloma; AML, acute myeloid leukaemia; MDS, Myelodysplastic syndrome; ALL, Acute lymphoblastic leukaemia; NHL, Non‐Hodgkin lymphoma; NSCLC, non‐small cell lung cancer; Others, sarcoma, glioblastoma, melanoma, bladder cancer.

Total delivery of anti‐cancer preparations increased by 26% between the two periods, from 1332 preparations with 2% for solid tumour drugs (*n* = 26) to 1684 with 8.5% for solid tumour drugs (*n* = 142) (*p* < 0.01).

**FIGURE 1:**
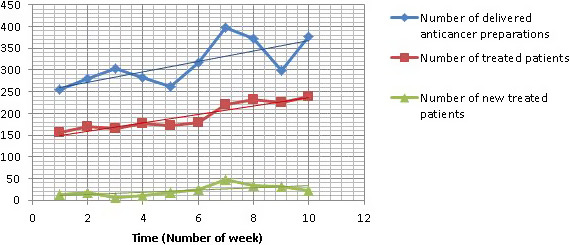
Evolution of activity in HAH each week in 2020 from 17 February to 26 April

The evolution of the weekly activity from 17 February to 26 April showed an increase of delivering preparations, of patients treated in HAH, and of new patients treated in HAH with a peak of new patients during the first week of period 2. Some patients were also cancelled due to COVID‐19 infection or confined in another area of France. No patient was readmitted from HAH to hospital. No nosocomial infection was reported after anti‐cancer injection in HAH during the period of study (Figure 1).

The panel of delivered drugs increased from 19 different drugs, with less than one‐third treating solid tumours, to 29 drugs during period 2 with half treating solid tumours (*p* < 0.01). Ten drugs increased during period 2, mainly bortezomib, carfilzomib, cytarabine for haematological malignancies, and eribulin, paclitaxel, pertuzumab, trastuzumab and trastuzumab emtansine for breast cancer. Subcutaneous injections of rituximab decreased by 45% during period 2 (Table 2). Five clinical departments prescribed a wider panel of drugs than during period 1, such as intravenous infusions of paclitaxel, daratumumab, carfilzomib, brentuximab, eribulin and trastuzumab emtansine. Three drugs were newly administered in HAH at the request of physicians and after agreement of HAH staff: belinostat for the treatment of relapsed or refractory peripheral T‐cell lymphoma after the first injection of each course in the outpatient clinic, durvalumab for non‐small cell lung cancer after at least one injection in the outpatient clinic and pertuzumab for HER2‐positive breast cancer after at least two injections received in the outpatient clinic to assess tolerance. No tolerance issues were reported at home.

**TABLE 2:** Anti‐cancer drug preparations delivered in HAH during the two periods

INN	Administration route	Number of preparations ‘period 1’ (%)	Number of preparations ‘period 2’ (%)	Evolution rate (%)
Arsenic Trioxide	IV	28 (2.1)	29 (1.7)	+3.6
Azacitidine	SC	874 (65.8)	920 (54.9)	+5.3
Bortezomib	SC	237 (17.8)	274 (16.3)	+15.6
Carfilzomib	IV	44 (3.3)	105 (6.3)	+138.6
Cytarabine	IV	63 (4.7)	113 (6.7)	+79.4
Daratumumab	IV	24 (1.8)	36 (2.1)	+50
Nivolumab	IV	4 (0.3)	9 (0.5)	+125
Paclitaxel	IV	2 (0.2)	11 (0.7)	+450
Rituximab	SC	20 (1.5)	11 (0.7)	−45
Trastuzumab	SC	7 (0.5)	72 (4.3)	+928.6
Vinorelbine	IV	9 (0.7)	15 (0.9)	+66.7
Others	SC or IV	17 (1.3)	81 (4.8)	+376.5
TOTAL		1329 (100)	1676 (100)	+26.1

INN, International Non‐proprietary Name; IV, intravenous; SC, subcutaneous; Others, Belinostat, Bendamustine, Bevacizumab, Bleomycin, Brentuximab Vedotin, SC Cytarabine SC, Dacarbazine, Doxorubicin, Durvalumab, Eribulin, Fluorouracile, Gemcitabine, Irinotecan, Pembrolizumab, Pertuzumab, IV Rituximab, Trastuzumab Emtansine, Vinblastine, Vincristine, Vindesine

* Chi‐squared test *p* < .01.

**FIGURE 2:**
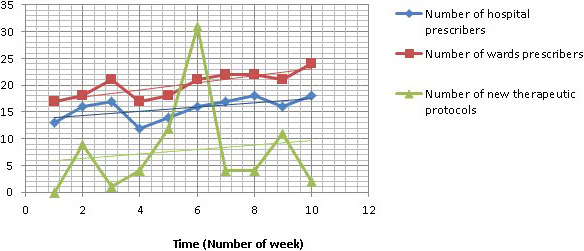
Evolution of new therapeutic protocols and prescribers in HAH each week in 2020 from 17 February to 26 April

The weekly evolution of new therapeutic protocols and new prescribers from 17 February to 26 April showed a frank increase during period 2, with a peak during the first week of second period (Figure 2). It mainly concerned protocols for solid tumours.

### 3.2 Adaptation of organisation and human resources during the pandemic

For the 15 clinical departments accustomed to using HAH, the usual organisation has been adjusted to avoid hospital visits: patients were treated at home for the entirety of their course, including the first day of each course. Blood test analysis were managed by a community lab 48 h before the day 1 of the course and each patient was called by the hospital physicians to assess his clinical status. Prescriptions were recorded in the CHIMIO® software as per usual for home administration. During the ongoing course, blood samples and clinical assessments were performed by the HAH nurse 48‐h before each injection at home. Five clinical departments increased the number of patients treated at home according to this organisation.

Regarding the usual clinical departments, five clinical wards widened the panel of drugs prescribed at home during period 2. Six departments modified the treatment pattern for patients currently being treated: the anti‐cancer drugs were administered at home in their entirety, including the first day of each course. It concerned patients treated for haematological malignancies with subcutaneous azacitidine and bortezomib, and patients treated for breast cancer with subcutaneous trastuzumab.

Three oncology departments and one radiotherapy department solicited HAH for the first time for anti‐cancer drug injections. This concerned mainly immunotherapy injections. After defining the needs, we adjusted the HAH organisation, with infusion time at home restricted to 30 minutes instead of the usual 2 h in order to care for as many patients as possible. Therefore, rituximab infusions and daratumumab infusions were kept in hospital for those aforementioned departments. The oncologists made their prescriptions directly into the HAH CHIMIO® software with secure remote access (Citrix®, Citrix Gateway) with support from HAH pharmacists. Blood samples were exclusively managed by community lab 48 h before each injection, including ongoing course and each patient was called by the oncologist to assess clinical status.

To face the increase of HAH activity, additional human resources were required with (i) a specialised pharmacist who used to work occasionally in the HAH Chemo Unit; (ii) technicians and pharmacy fellows for compounding the anti‐cancer preparations, who had formerly worked in departments whose activity had decreased during the pandemic; (iii) HAH coordinating nurses for patient admissions with a switch from part‐time to full‐time, and the return of recently retired nurses; (iv) community nurses who had used to work occasionally for HAH; (v) hospital workers for handling and (vi) delivery men who had been freed up by the drop in other sectors. Travel times for the nurses between two patients and for drug delivery was improved thanks to the reduction of road traffic during the population's lockdown.

## 4 DISCUSSION

Our study related how HAH allowed anti‐cancer injection to continue during the pandemic without delays to treatment, to decrease unnecessary patient travel to hospital and consequently to decrease the COVID‐19 transmission risk and how follow‐up of patients during the pandemic was possible thanks to the adaptation of HAH, community health care and clinical ward organisation.

Care reorganisation during the COVID‐19 pandemic benefited mainly younger, more often female patients with solid tumours. Haematological malignancies such as multiple myeloma, myelodysplastic syndrome and acute myeloid leukaemia remained most frequent in HAH even if they showed a slight proportional downward trend during period 2. Our HAH structure is usually involved in the patient's pathway for long‐term treatment with very close injections of drugs like azacitidine or bortezomib to avoid patient journeys. This organisation was enhanced during the lockdown period with the first injection of each regimen in HAH for haematological malignancies as older patients and people with health conditions may get more serious symptoms from COVID‐19. The number of patients treated with carfilzomib also increased as multiple myeloma increases the risk of infectious disease due to the patients’ immunocompromised state, older age and comorbidities.^16^ However, during the COVID‐19 pandemic, it was patients with solid tumours, especially breast cancer, for whom the shift from hospital to HAH was most frequent. This could be explained by the development of well‐tolerated immunotherapies such as trastuzumab emtansine, trastuzumab and pertuzumab becoming more easily feasible at home, due to the pre‐existing bond between clinical departments and HAH for women’ post‐operative follow‐up or post‐chemo follow‐up, or for patients with other cancers treated at home with the same drugs such as paclitaxel. Only patients with non‐Hodgkin lymphoma usually treated in HAH with subcutaneous rituximab were fewer during the lockdown period as their programme of injections every 2 months was lengthened. To our knowledge, HAH APHP provides the widest panel of drugs administered at home described in the literature, though not all anti‐cancer drugs are suitable for HAH administration depending on their stability, their infusion time and their tolerance. In addition, we had to reduce the infusion time usually accepted in our structure during this lockdown period to treat the maximum of patients at home. Therefore, we could not increase the number of patients with multiple myeloma treated with infusion of daratumumab.

The shift from hospital delivery to HAH led to human and logistical challenges, as the increase of activity in HAH was consistent with a sustained workload, especially at the beginning of the lockdown period, with the addition of new prescribers, and the need to meet the prescribers’ demands within a short time. It was possible to set it up quickly, as our HAH structure is used to work with community health care and hospital. Physicians and HAH should continue to adapt and evolve their practices. To decrease the HAH workload, we asked the oncologists to prescribe directly into the HAH CHIMIO® software as the time was too short to set up interfaces between the CHIMIO® softwares and the workload was too heavy for our HAH coordinator doctor to rewrite all prescriptions in the HAH CHIMIO® software. Our organisation allowed us to treat more patients per week for their cancer while decreasing the pressure on hospital health services and freeing up hospital nurses, who could potentially work in intensive care unit during the pandemic period. Community staff represented additional humans resources, as our HAH was used to work with community nurses and labs, leading to a specific organisation with trained staff in sufficient numbers who could be mobilised in a short time in case of need.

However, HAH avoided patients’ presence at hospitals and allowed the management of patients with cancer, including the injection of anti‐cancer drugs, at home. When lockdown stops patients will return to hospital as they require medical examination by specialist physicians, but further treatment may continue at home. It will be useful to take stock with physicians and check if patients continue in HAH. For the new clinical departments, the organisation requires the instalment of a computer interface to avoid physicians juggling between two softwares.

The offer and skills of HAH should be promoted as routine, as our study showed that administration at home could be quickly implemented in case of emergency in a new clinical department, or its cancer activity increased if a suitable organisation already exists. The HAH’s organisation required flexibility and skilled community nurses who occasionally worked in HAH. HAH allowed the management of patients for the provision of safe and effective cancer care during the pandemic. Our current intervention measures in HAH related to COVID‐19 have been reported in the hope that it may provide a reference for future studies, as this organisation could be improved in the future with the use of telemedicine instead of phone calls for clinical status assessment.

We should acknowledge the limits of this experiment. The offer of HAH is territorially unevenly distributed in France and around the world.^14^ Cancer activity is heterogeneous within the HAH structures, with restricted cancer activity or a limited panel of the eligible drugs, suitable for treating the most widespread needs. Despite additional human resources, we limited the use of HAH health care for the blood testing connected with clinical status collection, and developed the use of labs and phone calls for clinical assessment by the physician himself to increase the anti‐cancer injection capacity. Moreover, one oncology department, which had initially requested the HAH service, did not finally prescribe anti‐cancer injections at home. This should be investigated to learn why HAH did not meet their needs or if another reason such as funding could explain it. Like the HAH structure, French hospitals are funded according to their activity. Consequently, the patient treated in HAH setting requires hospital medical time which, with this funding system, is not paid at the hospital which may result in an obstacle to its use.

As a cancer management strategy, HAH should be considered as a real alternative to hospital administration and for avoiding systematic hospital visits for anti‐cancer drug administration, whether during the lockdown or long term. HAH should be included in guidelines for cancer administration and not restricted to follow‐up or oral treatment (4,6,8).

## 5 CONCLUSIONS

This study described how HAH could be used to decrease unnecessary travel to hospital and allow further anti‐cancer treatments and follow‐up from home, particularly during the pandemic period. Moreover, as cancer is becoming a long‐term disease, along with advances in novel targeted treatments and the increase of cancer patients, with the associated financial constraints, it is necessary to rethink the classical cancer management scheme and develop alternative models of health service delivery, such as home programmes to manage this public health issue in both the short and the long term.

## CONFLICT OF INTEREST

None declared.

## Ethics approval and consent to participate

All patients provided written informed consent to be treated at home and agreed to the reuse of their health data anonymously collected during the care in our structure for research purposes before admission in HAH. The study has been submitted to the ethic committee CERAPHP5, but not required approval as the study was monocentric and retrospective, patients were treated according to the standard clinical practice including no additional interventions and data were anonymous with no identifiable patient information. However, the study has been recorded to the general register of Assistance Publique des Hôpitaux de Paris according to the data protection legislation (n° 20200715132522).

## AUTHOR CONTRIBUTIONS

All authors made substantive contributions to the development of this study. BMM wrote the protocol and manuscript, provided, analysed, and interpreted data. BMM, AZ, MCP, MLBP, FA, JG, IM contributed to the patients’ care management. BMM, JCC, VC, LH, EB, TL, LN contributed to HAH organisation. MDS and PA commented critically on the manuscript and contributed to study design and manuscript preparation.

## Data Availability

The data sets used and/or analysed during the current study are available from the corresponding author on reasonable request.
